# Dosage Compensation and Gene Expression of the X Chromosome in Sheep

**DOI:** 10.1534/g3.118.200815

**Published:** 2018-11-27

**Authors:** Jingyue (Ellie) Duan, Kaleigh Flock, Nathanial Jue, Mingyuan Zhang, Amanda Jones, Sahar Al Seesi, Ion Mandoiu, Sambhu Pillai, Maria Hoffman, Rachel O’Neill, Steven Zinn, Kristen Govoni, Sarah Reed, Hesheng Jiang, Zongliang (Carl) Jiang, Xiuchun (Cindy) Tian

**Affiliations:** * Department of Animal Science,; ** Department of Computer Science,; †† Department of Molecular and Cell Biology, and University of Connecticut, Storrs, CT, 06269,; † School of Natural Sciences, California State University, Monterey Bay, Seaside, CA 93955,; ‡ Laboratory Animal Center, Guangxi Medical University, Nanning 530021, China,; § Smith College Department of Computer Science, Northampton, MA 01063,; ‡‡ College of Animal Science and Technology, Guangxi University, Nanning 530004, China, and; §§ School of Animal Science, Louisiana State University, Baton Rouge, LA 70803,

**Keywords:** Ohno’s hypothesis, X chromosome upregulation, Maternal nutrition, Ovine

## Abstract

Ohno’s hypothesis predicts that the expression of the single X chromosome in males needs compensatory upregulation to balance its dosage with that of the diploid autosomes. Additionally, X chromosome inactivation ensures that quadruple expression of the two X chromosomes is avoided in females. These mechanisms have been actively studied in mice and humans but lag behind in domestic species. Using RNA sequencing data, we analyzed the X chromosome upregulation in sheep fetal tissues from day 135 of gestation under control, over or restricted maternal diets (100%, 140% and 60% of National Research Council Total Digestible Nutrients), and in conceptuses, juvenile, and adult somatic tissues. By computing the mean expression ratio of all X-linked genes to all autosomal genes (X:A), we found that all samples displayed some levels of X chromosome upregulation. The degrees of X upregulation were not significant (P-value = 0.74) between ovine females and males in the same somatic tissues. Brain, however, displayed complete X upregulation. Interestingly, the male and female reproduction-related tissues exhibited divergent X dosage upregulation. Moreover, expression upregulation of the X chromosome in fetal tissues was not affected by maternal diets. Maternal nutrition, however, did change expression levels of several X-linked genes, such as sex determination genes *SOX3* and *NR0B1*. In summary, our results showed that X chromosome upregulation occurred in nearly all sheep somatic tissues analyzed, thus support Ohno’s hypothesis in a new species. However, the levels of upregulation differed by different subgroups of genes such as those that are house-keeping and “dosage-sensitive”.

In mammals, deviation from diploidy may induce detrimental consequences (Birchler *et al.* 2005). For example, gene duplications or deletions can induce cancer (Giam and Rancati 2015), and chromosome monosomy or trisomy usually causes fetal lethality (Burgess *et al.* 2014). Mammalian males, however, are monosomic for the X chromosome, yet do not suffer from the deleterious effects of X monosomy (Chen *et al.* 2014). This is likely the result of a still debated mechanism of X chromosome dosage compensation (Pessia *et al.* 2014; Mank *et al.* 2014; Veitia *et al.* 2015; Graves 2016). Susumu Ohno hypothesized that upregulation of the X-linked genes in the heterogametic sex (XY) would be necessary to maintain their expression to the levels of the diploid autosomes (Ohno 1966). This solved the dosage imbalance of X-linked genes in males, yet subjected females to quadruple levels of X expression. Another mechanism, X chromosome inactivation (XCI), the inactivation of one of the two X chromosomes in every cell of the female, balances the X chromosome gene dosage between males and females (Lyon 1961). Both X chromosome upregulation and XCI are necessary components of the X chromosome dosage compensation in mammals (Ohno 1966).

Although XCI has been characterized in many species (Goto and Monk 1998; Xue *et al.* 2002; Deakin *et al.* 2009; Lee 2011; Livernois *et al.* 2012; Sahakyan *et al.* 2018), verification of X chromosome upregulation has not been conducted until chromosome-wide expression analysis became possible (Pessia *et al.* 2014). X chromosome upregulation has been studied using data from microarray (Gupta *et al.* 2006; Nguyen and Disteche 2006) and RNA sequencing (RNA-seq) (Xiong *et al.* 2010; Deng *et al.* 2011; Lin *et al.* 2007, 2011, 2012; Pessia *et al.* 2012) by computing the mean or median expression ratio of all X-linked genes to all autosomal genes (X:A). An X:A ratio of 1.0 or greater implies the doubling of X-linked gene transcription from the single active X, indicating complete compensatory upregulation. An X:A ratio of 0.5 indicates that expression levels of X-linked genes is half of those of the autosome pairs, suggesting no compensatory upregulation. When an X:A ratio falls between 0.5 and 1, it is termed partial compensation (Deng *et al.* 2011). A full compensatory upregulation of X-linked genes has been recently observed only in “dosage-sensitive” genes in eutherian mammals (Julien *et al.*, 2012; Lin *et al.*, 2012; Pessia *et al.*, 2012). These genes usually code for proteins complexes with structural, regulatory and housekeeping functions (Kondrashov and Koonin 2004; Birchler 2012; Pessia *et al.* 2012).

XCI and X chromosome upregulation have been studied in mice and humans, but such investigations lag behind in domestic species (Disteche 2012). XCI has been shown in sheep (Luciani *et al.* 1979), but little is known about its onset and no information is available on X dosage compensation. With completion of the ovine genome sequencing (Jiang *et al.* 2014) and the advancement of RNA-seq technology (Wolf and Bryk 2011), a number of RNA-seq datasets in sheep somatic tissues are now available for X chromosome upregulation studies.

The sheep has been frequently used as a model for human pregnancy and fetal development (Barry and Anthony 2008). Poor maternal nutrition, either over- or restricted-feeding, has been shown to alter gene expression in fetal tissues (Du *et al.* 2011; Pillai *et al.* 2016; Duan *et al.* 2018). Changes in DNA methylation is likely involved because restrictedly nourished ewes carried fetuses with altered DNA methyltransferase in the hypothalamus (Begum *et al.* 2012). Similarly, human metastable epialleles, which are variably expressed in genetically identical individuals, have also been persistently changed epigenetically by maternal nutrition in early pregnancy (Dominguez-Salas *et al.* 2014). These findings that maternal diet alters fetal epigenetics are of particular interest because XCI and X chromosome upregulation are epigenetically regulated processes (Goto and Monk 1998). However, the effects of maternal nutrition on X chromosome dosage compensation and X-linked gene expression have yet to be studied in the sheep, an important species for both agriculture and human medicine.

Using data generated by us (GSE111306) (Duan *et al.* 2018) and two additional RNA-seq datasets, PRJEB6169 (Jiang *et al.* 2014) and PRJNA254105 (Brooks *et al.* 2015), we were able to achieve the first comprehensive evaluation of X chromosome upregulation in sheep. Furthermore, we also investigated the effects of different maternal diets on the expression of X-linked genes. Our hypothesis was that X chromosome upregulation in the sheep would be partial, similar to that in the bovine as reported by us (Duan *et al.* 2016) and others (Ka *et al.* 2016). We further hypothesized that different maternal diets would alter the expression levels of X-linked genes in ovine fetal tissues.

## Materials and Methods

### Experimental design and RNA sequencing

Animal protocols, tissues collection, and RNA sequencing library preparation were described in Pillai *et al.* (2017) and Duan *et al.* (2018). Briefly, 12 pregnant ewes were individually housed and randomly assigned to control- (100% NRC requirement, Con, n = 4), overfed- (140%, Over, n = 4) or restricted- (60%, Res, n = 4) diets calculated by the National Research Council requirement for total digestible nutrients for a ewe pregnant with twins (Pillai *et al.* 2016). The ewes remained on their respective diets until day 135 of gestation when they were killed. Fifteen fetuses, control (n = 7), overfed (n = 4) and restricted (n = 4) were included in this study. Full organ of brain, kidney, and lung were collected, flash-frozen in liquid nitrogen and stored at -80° until RNA extraction.

RNA was extracted from fetal brain, kidney, and lung using TRIzol (Invitrogen, Grand Island, NY) according to the manufacturer’s instructions. Library preparation was carried out using TruSeq RNA library prep kit (Illumina, RS-122-2001, RS-122-2002) and quantified using real-time PCR. Agilent 2100 Bioanalyzer (Agilent) was used to assess the size distribution and to determine the RNA integrity number (RIN). All RNA samples for sequencing had RIN values greater or equal to 7 (Duan *et al.* 2018). The sequencing was performed on Nextseq 500 System (Illumina) with 75 bp paired-end reads in three sequencing runs. Overall, we obtained 2,149 million raw sequencing reads that passed filtering from three sequencing runs of 45 fetal tissue samples. The raw read dataset has been uploaded to GEO database with the accession number GSE111306.

### Additional RNA-seq datasets

In addition to the RNA-seq data described above, two additional RNA-seq datasets were downloaded from Sequence Read Archive (SRA; http://www.ncbi.nlm.nih.gov/sra) under the accession numbers PRJNA254105 (Brooks *et al.* 2015) and PRJEB6169 (Jiang *et al.* 2014). PRJNA254105 included whole conceptuses at day 14 of gestation. PRJEB6169 contained data from adult and juvenile (6-10 months) heart, brain, liver, biceps femoris, rumen, female and male specific tissues, including the cervix, ovarian follicles, ovary, uterus, corpus luteum, testes, and placenta and membranes.

### RNA-seq data trimming, mapping and assembly

Sequence adapter and quality trimming were conducted using Sickle v1.33 (Joshi and Fass 2011) with the parameters Q score ≥ 30 and length ≥ 20 (-q30, -l20). RNA-seq reads were checked using FastQC v0.11.3 (Andrews 2010) for quality control. Filtered RNA-seq reads from fetal tissues of day 135 of gestation were aligned to the sheep reference genome Oar_v4.0 using Hisat2 v2.0.5 (Kim *et al.* 2015). The mapping rates of all datasets are summarized in Table S1. The average mapping rate for our data are 90% with 19,846,496 reads mapped to the genome, whereas the additional datasets had an averaged 75% mapping rate with 12,854,507 reads aligned.

Aligned reads for each tissue from all three datasets were assembled using IsoEM v1.1.4 (Nicolae *et al.* 2011). The mRNA level of each gene was estimated by log_2_-transformed transcripts per kilobase million (TPM) within each dataset and quantified using IsoEM (version 1.1.4; Nicolae *et al.*, 2011). TPM normalizes for gene length first and then for sequencing depth. This was preferred to RPKM/FPKM because it normalizes among transcriptome sizes of different samples and allows more appropriate comparisons of gene expression across samples (Soneson *et al.* 2015). Gene expression levels in TPM were log_2_-transformed to minimize variations. Expressed genes were defined as TPM ≥ 1 (Clark *et al.* 2017). A total of 7,166 genes were expressed among all tissues and defined as “dosage sensitive” genes (Sangrithi *et al.* 2017). Genes in the pseudoautosomal regions (PARs) of the sex chromosomes were obtained from Ruminant PARs annotation by Raudsepp and Chowdhary (2015).

### Dosage compensation calculation

A total of 20,519 genes are in the sheep genome and assigned to each chromosome. The X:A ratio was calculated as the Relative X Expression (RXE); the difference between the log_2_-transformed mean TPM values of the X chromosome and autosomes (A), using the formula below, where X-linked and autosomal genes were expressed as *x* and *a*, respectively:

$$RXE=\log_{2}\left(\frac{x}{a}\right)=\log_{2}x-\log_{2}a$$

An RXE ≥ 0 represents a full up-regulation of X. An RXE between 0 and -1 indicates partial X chromosome upregulation. An RXE of -1 indicates a lack of X chromosome upregulation.

We also calculated the relative expression of each autosome pair (RGE) over all other chromosomes (excluding mitochondria and the Y chromosome which are not annotated in sheep). The RGE value was used to determine if the expression of the X chromosome deviated from the normal range of expression by the autosomes and if a particular autosome pair is more/less expressed than the rest of the chromosomes. The RGE was calculated using the following formula:

$$RGE=\log_{2}\left(\frac{a_{i}}{a_{n-i}}\right)=\log_{2}a_{i}-\log_{2}a_{n-i}$$

Where *i* represents a particular pair of autosomes, *n* represents all autosomes. *n-i* represents all autosomes excluding the autosome *i*. If the RGE of an autosome pair was greater than or equal to 0, it represents upregulation of that autosome pair. An RGE between 0 and -1 indicates downregulation.

The boxplots of RXE and RGE were generated in R (R Development Core Team 2008) using ggplot2 package (Wickham 2009). In these plots the lower and upper hinges encompass the 25^th^ and 75^th^ percentile of the data. The distance between the hinges is the inter-quartile range (IQR). The lower and upper whiskers extending from the hinges represent values no further than 1.fivefold of the IQR or within 95% confidence interval. Outliers were plotted individually beyond the end of whiskers (McGill *et al.* 1978) and labeled with numbers of the corresponding chromosomes.

### Differentially expressed X-linked genes across maternal nutrition

Differentially expressed genes (DEGs) between Con and Over or Con and Res were determined using IsoDE version 2 (Al Seesi *et al.* 2014; Mandric *et al.* 2017). IsoDE2 is based on 200 bootstrap replicates where sampling from the original data were performed with replacement and stratified by the group variables (Al Seesi *et al.* 2014). Bootstrapping (Efron and Tibshirani 1994) is advantageous because it offers a reliable solution to the lack/low replicates and allows distinction between biological differences and technical variability or noise. Bootstrapping method is simple to apply and does not require any distribution assumptions. In each comparison, a gene was deemed differentially expressed if it showed log_2_ fold change (FC) > 1 between two treatments and significantly different (P-value ≤ 0.05). The X-linked DEGs (Table S2) were a sub-group of the total DEGs from all chromosomes.

### Gene ontology analysis

A Gene Ontology (GO) classification was conducted using DAVID 6.8 (Huang *et al.* 2009b, 2009a). GO categories with P-value ≤ 0.05 were considered significantly overrepresented. The pie plot of “dosage sensitive” genes categorized by protein functions was made in PANTHER classification system (Mi *et al.* 2013).

### Data Availability Statement

The datasets analyzed during the current study are available in the Gene Expression Omnibus and BioProject:

GSE111306: https://www.ncbi.nlm.nih.gov/geo/query/acc.cgi?acc=GSE111306

PRJNA254105: https://www.ncbi.nlm.nih.gov/bioproject/?term=PRJNA254105

PRJEB6169: https://www.ncbi.nlm.nih.gov/bioproject/?term=PRJEB6169

Supplemental material available at Figshare: https://doi.org/10.25387/g3.7221467.

## Results

### X chromosome upregulation in ovine major organs and reproductive tissues

**Figure 1:**
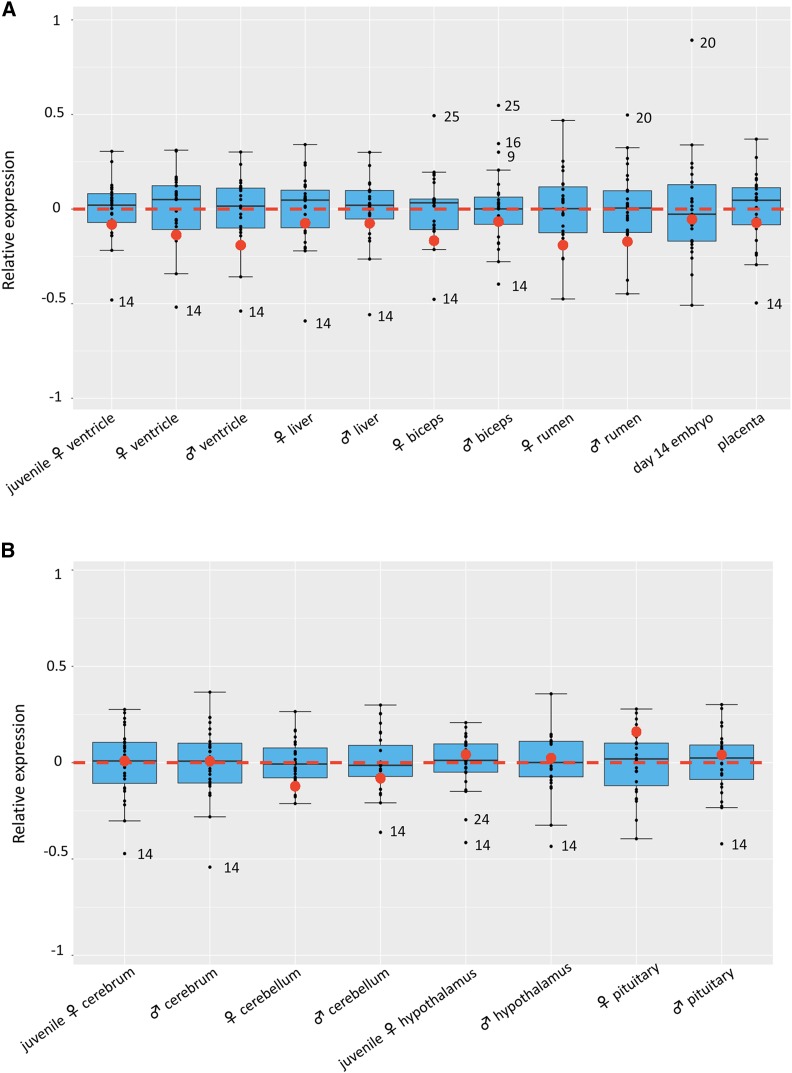
Boxplots of log_2_-transformed relative expression of the X chromosome (RXE) and each autosome pair (RGE) in major tissues (A) and brain (B) of juvenile and adult sheep. Red dots: mean RXEs for all replicates within a tissue type. Black dots: mean RGEs for each autosome pair. Numbers by black dots: autosomes whose RGEs fell outside of the expression quartiles for the tissue. Red dotted line: the border for complete (above line) and incomplete (below line) dosage compensation. The X:A ratio was calculated as the Relative X expression, RXE = log_2_ (X) − log_2_ (A), the difference between the log_2_-transformed mean TPM values of X and A. An RXE value of 0 means the expression of X and autosome is equal, suggesting X dosage compensation. Positive and negative RXE values indicate complete and incomplete dosage compensation, respectively. An RXE of -1, however, represents the lack of X dosage compensation. RGE of each autosome pair over all other chromosomes was used to evaluate the deviation of X expression to autosomes.

We found that the liver, muscle, rumen, heart of juveniles and adults, conceptuses and placenta and membrane at day 14 of gestation displayed partial X chromosome upregulation with RXE values ranging from -0.19 to -0.05, and an overall average RXE of -0.12 **(**Figure 1A**)**. Interestingly, all RXE were much closer to 0 than to -1, indicating a substantial amount of dosage compensation across the entire X chromosome. The upregulation, however, appeared to be more pronounced in the brain. The RXE ranged from -0.12 to 0.16 in the cerebrum, cerebellum, hypothalamus, and pituitary **(**Figure 1B**)**, suggesting complete X chromosome upregulation with the exception of the cerebellum. No significant difference (P-value = 0.74, by student *t*-test) was observed between males and females in the same tissue, demonstrating that X chromosome upregulation occurred similarly in both sexes, despite of the difference in the number of X chromosomes. Moreover, the RXE values fell within 1.5 times of the interquartile ranges (25–75% of the data) of RGEs of autosome pairs for all examined tissues (Figure 1 A and 1B), suggesting the single active X chromosome in somatic tissues balanced its gene transcription outputs with those of the autosome pairs.

**Figure 2:**
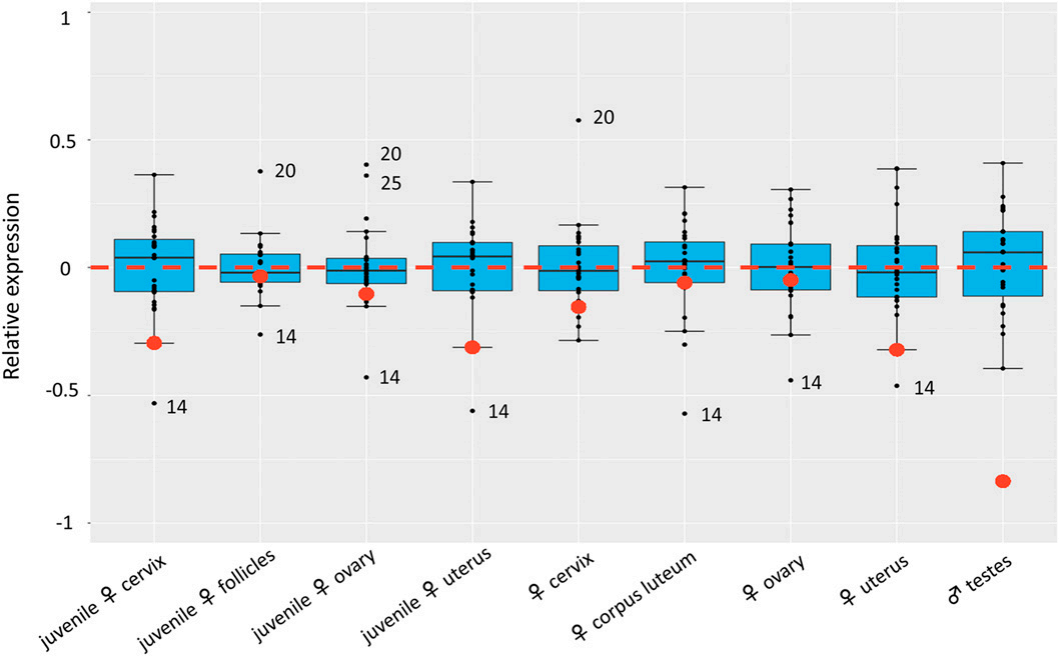
Boxplots of log_2_-transformed relative expression of the X chromosome (RXE) and each autosome pair (RGE) in female- and male-specific tissues. Red dots: RXEs for all replicates within a tissue type. Black dots: RGEs for each autosome pair. Numbers by black dots: autosomes whose RGEs fell outside of the expression quartiles for the tissue. Red dotted line: the border for complete (above line) and incomplete (below line) dosage compensation. The X:A ratio was calculated as the Relative X expression, RXE = log_2_ (X) − log_2_ (A), the difference between the log_2_-transformed mean TPM values of X and A. An RXE value of 0 means the expression of X and autosome is equal, suggesting X dosage compensation. Positive and negative RXE values indicate complete and incomplete dosage compensation, respectively. An RXE of -1, however, represents the lack of X dosage compensation. RGE of each autosome pair over all other chromosomes was used to evaluate the deviation of X expression to autosomes.

Partial X chromosome upregulation was also observed in juvenile and adult female reproduction-related tissues. These included the cervix, ovarian follicle, ovary, uterus, and corpus luteum. The overall averaged RXE was -0.19 and -0.15 for juvenile and adult female tissues, respectively **(**Figure 2**)**. Among these, juvenile follicles, adult ovaries and corpora lutea had the greatest RXE values. However, a different pattern was observed in the male specific reproductive tissues studied. The testes exhibited an average RXE of -0.84, or a near lack of X chromosome upregulation (*i.e.*, RXE = -1; Figure 2).

We also observed that a number of autosome pairs had either greater than or less than the overall averaged gene expression. Chromosome 14, for example, was very “quiet” in gene expression at the chromosomal level, falling outside of the 1.5 interquartile ranges of RGEs of the other autosome pairs in many tissues (Figure 1 A). Conversely, Chromosomes 20 and 25 were “active” in many tissues with high RGE values (greater than 0.5). Interestingly, the expression activity was negatively correlated (*r* = -0.94) with the numbers of expressed genes (TPM ≥ 1) on these chromosomes. With an averaged 751 expressed genes, Chromosome 14 was less active (average RGE=-0.44) than Chromosomes 20 (average RGE = 0.22) and 25 (average RGE = 0.25) with 416 and 202 expressed genes, respectively. Rather, expression activity of these chromosomes may be related to the functions of genes that they contain. We therefore analyzed the gene ontology (GO) terms of lowly (1≤ TPM < 50) expressed genes on Chromosomes 14, 20, and 25. The GO terms for Chromosome 14 included regulation of DNA-templated transcription, which corresponds to the reduced expression of transcription factors in most of tissues (Vaquerizas *et al.* 2009). On the other hand, the major GO categories of highly expressed genes (TPM > 100) on Chromosomes 20 and 25 were enriched in nucleosome assembly and sarcomere organization (Table S3). These terms corresponded to greater activities of Chromosome 20 in early conceptuses and Chromosome 25 in bicep muscles (Figure 1A).

### X chromosome upregulation in ovine fetuses under different maternal nutrition

**Figure 3:**
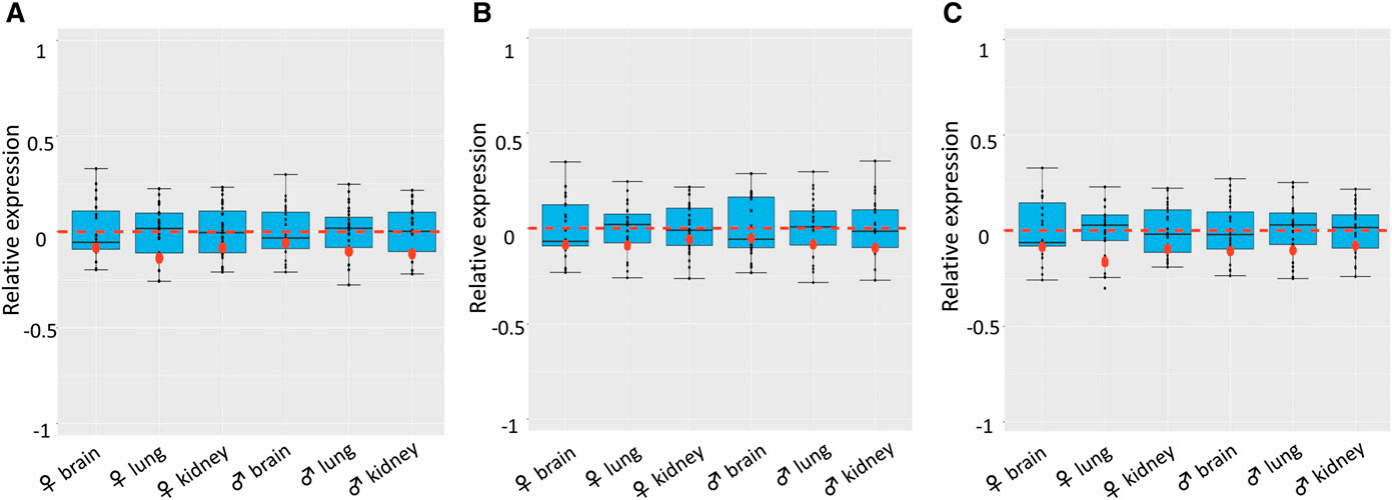
Boxplots of log_2_-transformed expression of the X chromosome (RXE) and each autosome pair by fetal tissues from mothers under different nutritional treatments: Control (A), Overfed (B) and Restricted (C). Red dots: RXEs for all replicates within a treatment group. Black dots: RGEs for each autosome pair. Numbers by black dots: autosomes whose RGEs fell outside of the expression quartiles for the tissue. Red dotted line: the border for complete (above line) and incomplete (below line) dosage compensation. The X:A ratio was calculated as the Relative X expression, RXE = log_2_ (X) − log_2_ (A), the difference between the log_2_-transformed mean TPM values of X and A. An RXE value of 0 means the expression of X and autosome is equal, suggesting X dosage compensation. Positive and negative RXE values indicate complete and incomplete dosage compensation, respectively. An RXE of -1, however, represents the lack of X dosage compensation. RGE of each autosome pair over all other chromosomes was used to evaluate the deviation of X expression to autosomes.

X chromosome upregulation in ovine fetuses at day 135 of gestation was not affected by maternal diet, either over- or restricted-nutrition (P-value = 0.59, 0.70, respectively, by Wilcoxon Rank Sums tests). Tissues (brain, kidney, and lung) of both male and female fetuses from mothers of all three treatment groups displayed partial dosage compensation. The RXEs of the three tissues ranged from -0.13 to -0.05, **(**Figure 3A**),** from -0.14 to -0.05 **(**Figure 3B**),** and from -0.16 to -0.08 **(**Figure 3C**)** for the Con-, Over- and Res-fed group, respectively. All RXE values fell within the RGE ranges, suggesting the single X upregulated on its expression to levels close to the autosome pairs. These observations indicate that different maternal nutrition did not affect the upregulation of the X chromosome in fetal tissues.

### X chromosome upregulation in different gene subgroups

**Figure 4:**
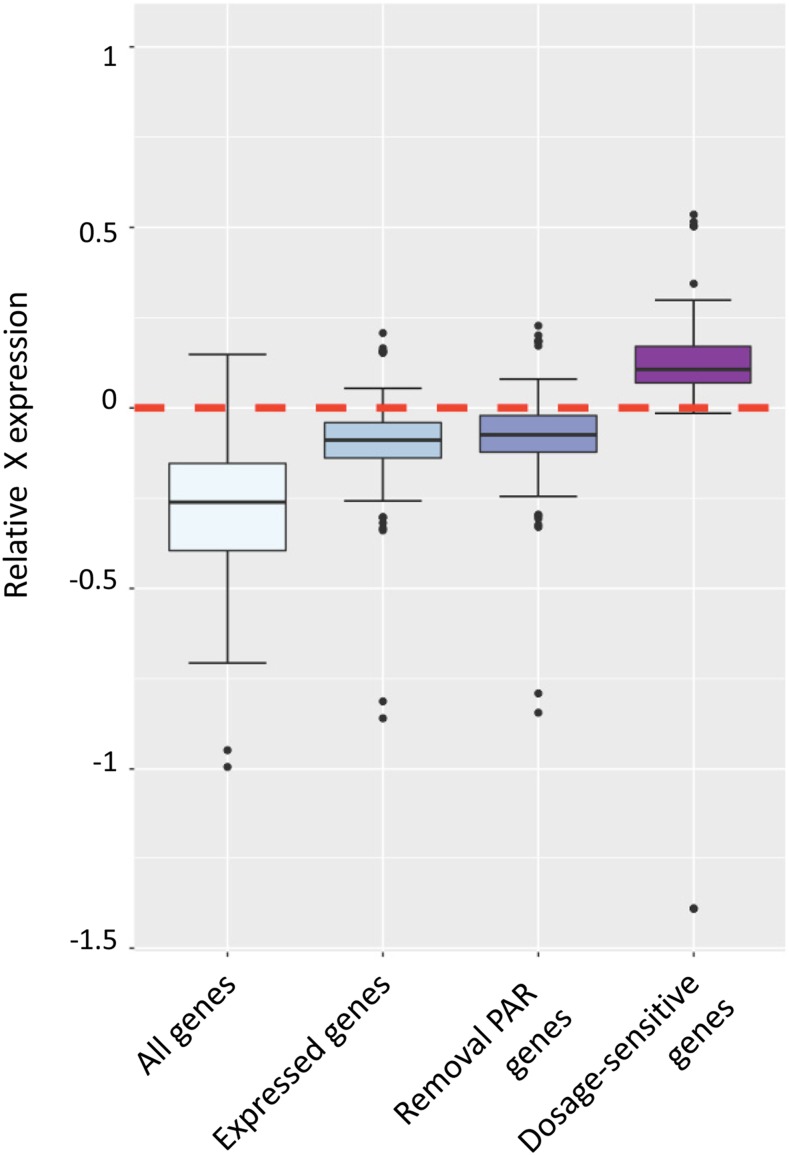
Boxplot of RXE values in the categories of “All genes”, “Expressed genes”, “Genes subject to XCI (removal of genes in PAR)” and “Dosage sensitive genes”. Red dotted line: the border for complete (above line) and incomplete (below line) dosage compensation.

We calculated the RXE values in the gene categories of “All genes”, “Expressed genes”, “Genes subject to XCI (removal of PAR genes)” and “Dosage sensitive genes” **(**Figure 4**)**. “All genes” included low- and non-expressed genes (TPM < 1), and had the lowest RXE values among the four subgroups of genes. The median of RXE in the “All genes” category was close to -0.5, indicating that when all X-linked genes were considered (including those with leaky expression), there was nearly no upregulation of the X chromosome. “Expressed genes” gave a partial X chromosome upregulation with a median RXE value close to 0, suggesting that this group contained genes of the X chromosome that were not subjected to X dosage compensation. Therefore, we removed the 14 genes located in ovine PAR. These genes have a homologous copy on the Y chromosome, and are not subjected to XCI. The RXE values without PAR were slightly increased, indicating that the PAR genes had lower expression. Moreover, we characterized another category - “Dosage sensitive genes”, which were ubiquitously expressed across all samples in our study and were mostly housing-keeping genes such as those involved in nucleic acid binding, cytoskeletal proteins and transferase (Figure S1 and Table S4). These “Dosage sensitive genes” had the highest median RXEs of greater than 0, corresponding to a full X upregulation. Taken together, our analysis of the four gene categories suggests that dosage regulation is highly related to gene functions.

### Effects of maternal nutrition on the expression of X-linked genes in ovine fetal tissues

A total of 1,228 X-linked genes were annotated in the current ovine genome (Jiang *et al.* 2014). Among these, 625 genes were expressed (TPM ≥ 1) by the three fetal tissues combined (Table S2). The mean number of expressed X-linked genes by each fetal tissue was calculated for each maternal nutrition group (Table 1). On average, 518.2 ± 14.7 out of 625 X-linked genes were expressed in the three organs. Specifically, the brain expressed the most genes (536.1 ± 14.3; control group), followed by the kidney (517.3 ± 9.2) and the lung expressed the fewest (506 ± 3.7). The numbers of the expressed X-linked genes were not significantly different (P-value > 0.05, by one-way ANOVA) across the maternal nutrition treatments (Table 1**)**. However, the levels of expression of the X-linked genes were affected by maternal nutrition. A total of 57 X-linked genes were differentially expressed among treatment groups. For example, two genes related to sex determination- *SOX3* and *NR0B1*-were down-regulated in fetal brains of the Over group (Table S5). The changes in sex-linked genes may provide a mechanism for the highly debated observation that skewed sex ratio was related to maternal nutrition (Mathews *et al.* 2008). The top eight X-linked DEGs (*PAGE4*, *S100G*, *SOX3*, *KCNE5*, *CLDN2*, *DUSP21*, *LOC105610402*, and *SLC6A14*) were summarized in Table 2 and were all expressed 8X (> 3 log_2_-Fold Change) more than that of the controls. Taken together, these expression data clearly demonstrated an effect of poor maternal nutrition on gene expression during fetal development.

**Table 1:** Mean numbers of expressed (TPM ≥ 1) X-linked genes in tissues of day 135 fetuses from control (n = 7), overfed (n = 4) and restricted (n = 4) mothers

	Treatments			
	Control	Overfed	Restricted	P-value
Brain	536.1 ± 15.4	531.8 ± 8.7	530.3 ± 9.2	0.73
Kidney	517.3 ± 9.9	514.5 ± 9.1	518.8 ± 2.2	0.77
Lung	506.0 ± 4.0	503.3 ± 7.4	502.0 ± 4.2	0.44

**Table 2:** Differentially expressed X-linked genes by tissues of ovine fetuses from mothers under control, overfed and restricted nutrition treatments

Comparison	Tissue	Gene	Expression in controls (TPM)	Expression in treated (TPM)	Log_2_ FC*
Con *vs.* Over	Brain	*PAGE4*	99.80	0.18	-∞
	Brain	*S100G*	33.62	0.46	-∞
	Brain	*SOX3*	1.23	0.15	-∞
	Kidney	*KCNE5*	1.71	0.27	-∞
	Kidney	*PAGE4*	1.46	0.86	−3.23
	Lung	*CLDN2*	0.26	6.36	4.71
Con *vs.* Res	Brain	*DUSP21*	0.08	1.04	5.21
	Brain	*LOC105610402*	1.04	0.00	-∞
	Brain	*S100G*	33.62	1.27	−5.20
	Lung	*SLC6A14*	1.57	0.10	−5.43

* Log_2_ FC: calculated by using bootstrapping; FC: fold change.

∞: infinity;

*PAGE4*: PAGE family member 4; *S100G*: S100 calcium binding protein G; *SOX3:* SRY-box 3; *KCNE5*: potassium voltage-gated channel subfamily E regulatory subunit 5; *CLDN2*: claudin 2; *DUSP21:* dual specificity phosphatase 21; *LOC105610402*: 60S ribosomal protein L17; *SLC6A14*: solute carrier family 6 member 14.

## Discussion

To our knowledge, this is the first study of X chromosome compensatory expression upregulation in sheep. We conclude that X chromosome upregulation was present, but largely partial. Additionally, X chromosome upregulation in fetal organs was not affected by the different maternal diets. While a number of species, both invertebrates and vertebrates, have been examined for their X:A ratios, whether X expression is globally upregulated is still highly debated [reviewed in (Gu and Walters 2017)]. Recent studies in therian mammals, including the human, mouse, bovine, and non-human primates mostly support the partial X chromosome upregulation conclusion with X:A ratio being close to 1 (Gu and Walters 2017; Duan *et al.*, 2016; Ka *et al.*, 2016). Our findings here contribute to the consensus of partial X chromosome upregulation in a new species.

The estimation of X:A ratios differs when different gene subgroups and different tissues are analyzed, thus resulting in completely different conclusions over Ohno’s hypothesis (Sangrithi and Turner 2018). Some of the low- and non-expressed genes in somatic tissues were found to be highly expressed in testis. These genes are more enriched on the X chromosome than on autosomes (Rice 1996; Deng *et al.* 2011; Disteche 2016). Therefore, when the analysis included low- and non-expressed genes, the estimation of X chromosome upregulation is biased. Our result showed that RXE was closer to -0.5 when “all genes” were included, while RXE was close to 0 when only expressed genes were used. These two different types of gene categorization and inclusion corresponded to the opposite findings by Xiong *et al.* (2010) and Deng *et al.* (2011). Additionally, dosage compensation requires both X chromosome upregulation and XCI. Not all genes on the inactive X, however, are silenced. A group of X-linked genes escape inactivation (Disteche *et al.* 2002; Berletch *et al.* 2011; Al Nadaf *et al.* 2012; Balaton and Brown 2016). These include all genes in PARs (Helena Mangs and Morris 2007) and a few in non-PAR regions of X (Tukiainen *et al.* 2017). As the homologous region of the mammalian sex chromosomes, genes on PARs are expressed from both the X and Y chromosomes (Vermeesch *et al.* 1997). However, we are not able to exclude any non-PAR genes from the group “subject to XCI” due to the lack of information on non-PAR genes that escape XCI from these regions in the ovine, we were only able to exclude PAR genes in the group of “Subject to XCI”. Of the 20 annotated genes in ovine PAR (Figure S2) (Raudsepp and Chowdhary 2015), 14 were expressed in our study. They were *P2RY8*, *DHRSX*, *ZBED1*, *CD99*, *XG*, *GYG2*, *ARSE*, *MXRA5*, *PRKX*, *NLGN4X*, *STS*, *PNPLA4*, *TBL1X*, and *GPR143*. They had relatively low expression levels, ranging in TPM from 1-50 while the average expression level of X-linked genes was 78.5 in TPM. Not much change in RXE values was found when the PAR genes were removed from the expressed group; possibly due their small number. Furthermore, our analysis showed a full compensatory upregulation of “dosage-sensitive” X-linked genes in sheep. This is in agreement with previous findings in the mouse and human by Ramsköld *et al.* (2009) and Sangrithi *et al.* (2017) who suggested that ubiquitous gene expression corresponded to the housekeeping function of dosage sensitive genes. It is likely that in order not to create limiting effects, gene products of this subgroup must be generated at comparable levels to those of the same pathways yet encoded by autosome pairs. Therefore, ubiquitously expressed genes are much more upregulated compared to other genes on the X chromosome.

There are a few exceptions to the general finding that ovine tissues underwent partial X chromosome upregulation. One exception is the brain, which had the greatest overall RXE values among all somatic tissues (RXE ranged from -0.12 to 0.16). This greater degree of X chromosome upregulation has also been observed in other species, including the human, mouse (Nguyen and Disteche 2006), old world monkeys, opossum, platypus, and chicken (Julien *et al.* 2012). The higher X chromosome upregulation is likely the result of both greater levels as well as numbers of expression of X-linked genes in the brain (Table1). During evolution, the X chromosome accumulated an excess of sex- and reproduction-related genes (Saifi and Chandra 1999). Greater expression of X-linked genes in the brain has been described as “the large X-chromosome effect” (Wu and Davis 1993), which was hypothesized to influence general cognitive ability, female mating choices and contribute to species diversification (Zechner *et al.* 2001). Therefore, it is expected that the brain would have a higher RXE.

Another exception to the overall X chromosome expression upregulation was seen in the sheep male reproduction-related tissues. The RXE was extremely low in sheep testes, corresponding to the observation of low X:A ratio in both the testes and spermatids in mice, indicating an X-specific partial repression in these cells (Nguyen and Disteche 2006). It was reported that the X:A ratio remained low in spermatogonia (Nguyen and Disteche 2006; Sangrithi and Turner 2018). Subsequently both the X and Y chromosomes become inactivated by meiotic sex chromosome inactivation during spermatogenesis (Manterola *et al.* 2009). This suppression of the X chromosome is likely the cause for the low X:A ratio.

Day 14 whole embryos, on the other hand, had an RXE value of -0.05 which was very close to full dosage compensation (RXE = 0). High X chromosome upregulation in early embryos could be a rebound after the release of repression of sex chromosomes in sperm. In the early embryos this release is necessary for X upregulation initiation (Wang *et al.* 2016). The expression of X chromosome was reported to be upregulated after the blastocyst stage which continued during 6.5 to 10.5 days post coitum development in mice (Nguyen and Disteche 2006). Mouse embryonic stem cells from both XX and XY embryos were also found to undergo X upregulation (Lin *et al.* 2011). Although XCI is known to operate in sheep fetuses (Luciani *et al.* 1979), little is known about its onset and regulation. In the bovine conceptuses, the onset of random XCI is found to have been established before day 14 (Bermejo-Alvarez *et al.* 2011). In the ovine, it is very likely that XCI has occurred by day 14 due to its shorter gestation (King *et al.* 1985; Stevens *et al.* 1990). It is therefore highly possible that the Day 14 ovine conceptuses had only one active X chromosome. The greater RXE value in Day 14 conceptuses thus may imply that the single active X chromosome just started its compensation process. This is consistent with the greater RXE values observed in Day 10-19 conceptuses in the bovine (Duan *et al.* 2017).

In summary, our comprehensive analyses of X chromosome dosage compensation suggest upregulation of gene expression from the single active X chromosome in most ovine tissues of both sexes.
